# A voxelwise approach to determine consensus regions-of-interest for the study of brain network plasticity

**DOI:** 10.3389/fnana.2015.00097

**Published:** 2015-07-28

**Authors:** Sarah M. Rajtmajer, Arnab Roy, Reka Albert, Peter C. M. Molenaar, Frank G. Hillary

**Affiliations:** ^1^Department of Mathematics, The Pennsylvania State UniversityUniversity Park, PA, USA; ^2^Department of Psychology, The Pennsylvania State UniversityUniversity Park, PA, USA; ^3^Department of Physics, The Pennsylvania State UniversityUniversity Park, PA, USA; ^4^Department of Human Development and Family Studies, The Pennsylvania State UniversityUniversity Park, PA, USA; ^5^Department of Neurology, Penn State Milton S. Hershey Medical CenterHershey, PA, USA

**Keywords:** fMRI, ROI selection, plasticity, modularity, label propagation, traumatic brain injury, hyperconnectivity

## Abstract

Despite exciting advances in the functional imaging of the brain, it remains a challenge to define regions of interest (ROIs) that do not require investigator supervision and permit examination of change in networks over time (or plasticity). Plasticity is most readily examined by maintaining ROIs constant via seed-based and anatomical-atlas based techniques, but these approaches are not data-driven, requiring definition based on prior experience (e.g., choice of seed-region, anatomical landmarks). These approaches are limiting especially when functional connectivity may evolve over time in areas that are finer than known anatomical landmarks or in areas outside predetermined seeded regions. An ideal method would permit investigators to study network plasticity due to learning, maturation effects, or clinical recovery via multiple time point data that can be compared to one another in the same ROI while also preserving the voxel-level data in those ROIs at each time point. Data-driven approaches (e.g., whole-brain voxelwise approaches) ameliorate concerns regarding investigator bias, but the fundamental problem of comparing the results between distinct data sets remains. In this paper we propose an approach, aggregate-initialized label propagation (AILP), which allows for data at separate time points to be compared for examining developmental processes resulting in network change (plasticity). To do so, we use a whole-brain modularity approach to parcellate the brain into anatomically constrained functional modules at separate time points and then apply the AILP algorithm to form a consensus set of ROIs for examining change over time. To demonstrate its utility, we make use of a known dataset of individuals with traumatic brain injury sampled at two time points during the first year of recovery and show how the AILP procedure can be applied to select regions of interest to be used in a graph theoretical analysis of plasticity.

## Background

The human brain has ~80 billion neurons each with between 100 and 1000 synaptic connections, making the task of modeling brain functioning the ultimate *big data* problem (Herculano-Houzel, 2009; Pakkenberg et al., 2003). While current functional brain imaging methods in humans (such as functional MRI) sample only a portion of this enormous network, including ~20–40 thousand voxels with time varying signals, data analyses remain computationally challenging. Because of this, most approaches under-represent the richness of the data available. In this paper we address an important methodological issue for neuroscientists aiming to examine developmental processes in neural networks and central to this issue is how investigators should partition the brain into functionally discrete regions. We focus our attention on bold oxygen level dependent functional MRI (BOLD fMRI) methods, so the unit of measurement for our purposes is the fMRI time series signal in each voxel although the concern regarding data parcellation applies to a number of methods. The goal is to develop a representative brain network, with regions-of-interest (ROIs) determined at the voxel level, in the service of examining change in the relationships between regions over time, i.e., plasticity.

To this end, we employ a whole-brain partitioning algorithm to divide the brain into functionally separate ROIs and then offer an approach for comparing these ROIs between time points: *aggregate-initialized label propagation (AILP)*. AILP permits retention of voxel-level information while affording comparisons between time points. To demonstrate how this approach can be used, we apply it to the study of whole-brain plasticity after traumatic brain injury (TBI) in resting state functional MRI (rsfMRI) data. We discuss here the available methods for data parcellation and we then turn to the focus of the current paper which is aggregation of distinct data parcellations in the service of studying plasticity. The following offers a brief treatment of each of these issues and how they are addressed in the current study.

### Region-of-interest selection

With respect to macro-scale efforts to understand brain organization there are several important caveats that must be emphasized to place this level of analysis in greater context within the neurosciences. First, there exists an entire world of connectivity complexities not observable using human brain imaging methods such as fMRI, including the connection subtypes and nature of the signal propagated (e.g., interneuronal inhibition vs. pyramidal cell excitation), variation in density of dendritic arborization even within cortical regions (e.g., frontal cortex; Jacobs et al., 2001), variation in connectional reciprocity between cell assemblies, and the possibility of distinct organization hierarchies within the brain (for review see Rockland, 2015). Moreover, human brain imaging work often presumes identical complexity in cell structure across cortex (i.e., commonly all voxels are created equal), which is an important simplification. As an example, there is strong evidence for a posterior to anterior gradient in increasing complexity in pyramidal cell organization and even distinct organization within prefrontal cortex (see Elston, 2000; Elston et al., 2005, 2011). So the contribution to the neurosciences made by human brain imaging resides at a different scale, providing the unique opportunity to examine the synchronization of coherent signals arising from large assemblies of cortical, subcortical, and cerebellar neurons, simultaneously.

In systems-level network science the most important early decision in network modeling is to determine the brain regions that will contribute to the network, which in network parlance are considered “nodes” (see Sporns, 2011). To avoid confusion during presentation of the findings in this study, we draw distinctions between network representations based on voxels and networks based on ROIs at each analytic step. For example, voxels entered into the network analysis during the first step during parcellation are voxel-nodes, and the term “node” is reserved specifically for graph theory/network analysis. **Table 2** provides definitions for key terms in each step of the analysis.

In systems-level studies of brain connectivity, the literature is replete with studies using seed-based and anatomical atlas-based techniques for ROI selection, but these approaches suffer from important shortcomings. Atlas-based approaches average the signal from heterogeneous voxels comprising each of the ROIs, leading to insensitivity to important regional interactions, while seed-based approaches involve investigator bias, do not sample signal outside the seeded region, and may include nuisance signal in the analysis (Power et al., 2012, 2014; Hallquist et al., 2013). Data-driven approaches such as ICA have proven to be a powerful alternative to these methods and there is now an impressive literature using spatially constrained ICA to examine between-group comparisons (e.g., Calhoun et al., 2001a,b; Allen et al., 2011; Calhoun and Adali, 2012). While it remains a valuable approach for some research questions, ICA typically requires investigator supervision, including assignment of the number of components within the component structure and selection of components as either viable or of no-interest (Allen et al., 2011; Smith et al., 2012).

In response to the need for more detailed analysis of functional brain space, there is a growing number of voxel-level parcellation methods aiming to determine ROIs without a priori information about their number or size (van den Heuvel et al., 2008; Yeo et al., 2011; Craddock et al., 2012; Blumensath et al., 2013). This problem is NP-hard, and not surprisingly, most approaches designed to solve it are computationally expensive, are initialization dependent, or require training of an objective function (see Honnorat et al., 2015). For now, the ideal approach largely depends upon the goal of the study.

### ROI aggregation and the study of network plasticity

Given the voxelwise options available, a primary concern for the current study is the unavoidable challenge *after parcellation* of guaranteeing that parcellation maps are comparable across time points. For voxelwise approaches, this issue is universal, irrespective of the specific parcellation routine chosen at the first step. Incompatibility between observations poses a dilemma for investigators interested in examining network plasticity during developmental processes such as learning, fatigue or even clinical recovery. To address this issue, investigators have analyzed all subjects and time points simultaneously (e.g., spatially constrained ICA; see Allen et al., 2011) or used only a single time point as the basis for ROI selection. However, both of these approaches have limitations. The challenge is to develop a reliable, efficient method that affords sensitivity to network plasticity and permits comparison of data between time points while preserving the quality of the original data (in our case voxel-level information) at the individual subject level. The goal of this study is to develop a procedure to examine system-level plasticity in fMRI datasets.

To achieve this goal we conduct whole-brain voxelwise analysis using *modularity* (Newman, 2006), a common measure of community structure borrowed from the graph theory literature (see **Table 2** for definitions). First, we constrain the modularity procedure using an established anatomical atlas (AAL, see below) because it is conservative, provides highly consistent results and allows for testing the label propagation method proposed here at the voxel level.

Following, we utilize a secondary aggregation and label propagation procedure that produces reliable regions consistent across time points for investigator comparison. In this second step, we apply a modified label propagation algorithm, based on the one originally developed and validated by Raghavan et al. (2007) for finding densely connected subsets of nodes in large graphs without prior knowledge of the number or size of resulting clusters. Specifically, we initialize the label propagation algorithm with an aggregate of the individual time point ROIs determined in the first step. We nickname this procedure AILP (aggregate-initialized label propagation). A primary advantage of AILP is that it permits concatenation of data sets from multiple time points while preserving information about the spatio-temporal characteristics of the BOLD signal at each voxel over time. The AILP procedure sorts based on the “identity” of local voxels determined at the first step (in our case the modularity), enforcing a rule that adjacent regions are grouped together as functional nodes. In this way, the AILP functions to cluster spatially contiguous voxels, which is consistent with the spatially embedded nature of brain organization, where local connectivity has higher probability than long-distance connections, which come at greater wiring and metabolic cost (see Mitchison, 1991; Cherniak, 1994; Ahn et al., 2006). Most importantly, the procedure for the AILP is straightforward, runs in near-linear time, and permits reliable data recombination across time points (see below).

#### Study goals

The primary goal of this study is to implement an efficient, computationally inexpensive approach for combining BOLD fMRI data sets between time points to examine plasticity. We make use of label propagation, an approach developed for examining community structure in large graphs. This approach uses network characteristics alone requiring little optimization or information *a priori* about the nature of the dataset being analyzed. To guarantee that network change is occurring we make use of a dataset where known recovery is occurring after brain injury (3 and 12 months post injury) and connectivity change has been observed using targeted analysis of specific subnetworks (Venkatesan et al., 2014).

## Materials and methods

### Subjects

Recruitment for the dataset analyzed here included 21 individuals diagnosed with moderate and severe traumatic brain injury. Of this group, 9 were excluded due to attrition or excessive head motion. This study included 12 people individuals sustaining moderate and severe traumatic brain injury and 12 healthy control adults (see Table 1 for demographic information). All subjects completed two separate sessions ~3 and 12 months following the resolution of post-traumatic amnesia, referred to as Time 1 and Time 2. A group of health control participants (HC) was also included to provide context for the expected network change over time using this approach. The HC sample completed two separate MRI sessions separated by ~3 months to provide context for natural variation in healthy brain connectivity (see Table 1). Each testing session included both MRI data collection and cognitive testing. Moderate and severe TBI was defined as a Glasgow Coma Scale (GCS) at time of injury of 3–8 was indicative of severe injury and a GCS of 9–12 was indicative of moderate injury (Teasdale and Jennett, 1974). Two subjects who received a GCS of 14 and one subject who received a GCS of 15 were included because their medical records indicated positive imaging findings showing damage to neural tissue or there was evidence of mental status change during their acute inpatient stay after the initial GCS assignment. Subjects were excluded if they were receiving treatment for concomitant injuries (e.g., orthopedic injuries or injury to the spinal cord) that would make it difficult for them to remain still and comfortable in the MRI environment.

**Table 1:** **Demographic descriptors and injury information**.

**Demographic**	**TBI Mean (sd)**	**Healthy controls Mean (sd)**	
Age (years)	27.9 (5.8)	35.6 (15.7)	*p* > 0.05
Education (years)	13.4 (2.5)	13.58 (1.93)	*p* > 0.05
Gender	6M, 6 F	7 F, 5 M	*p* > 0.05
GCS	6.73 (4.3)^T^	na	na
Time-post injury (months)	3.25 (0.97)	na	na
Time-between scans (months)	8.78 (2.83)	3.58 (1.56)	na

GCS, Glasgow Coma Scale; ^T^GCS, when available; when not available, inclusion based upon positive acute CT finding; sd, standard deviation.

Research was approved by the institutional review board and the Pennsylvania State University Office of Research Protections. Individuals were included in the study demonstrated some level of cognitive impairment. If an individual retained the ability to sign medical documents and/or function independently, then consent was accepted; if the individual was not functionally independent, then a caregiver's signature was required in addition to the subject's signature of assent.

### Functional imaging data acquisition

Participants were scanned using one of three MRI machines, including a Philips Achieva 3T scanner in the Department of Radiology at Hershey Medical Center, Hershey, PA (*n* = 7) and two Siemens Magnetom Trio 3T scanners (Social, Life, and Engineering Sciences Imaging Center at the Pennsylvania State University in University Park, PA (*n* = 5); Department of Radiology at Hershey Medical Center in Hershey, PA, *n* = 12). For repeat scanning, all subjects were scanned on the same MRI machine across time points. Prior to scanning subjects were made aware of the importance of minimizing motion within the scanner. The sample here is a select group of subjects from prior work (Venkatesan et al., 2014).

#### Data acquisition parameters

Anatomical images with a spatial resolution of 1.0 × 1.0 × 1.0 mm were acquired using an MPRAGE sequence: 2000 ms/2.03 ms/9° (repetition time (TR)/echo time (TE)/flip angle (FA), 256 × 256 mm^2^ field of view (FOV), and 256 × 256 acquisition matrix with 1 mm slices. Echo planar imaging (EPI) was used to examine the blood oxygen level dependent response for functioning imaging. Imaging parameters for EPI were 2000 ms/30 ms/90° (TR/TE/FA), 240 × 240 mm^2^ FOV, and 80 × 80 acquisition matrix with 4 mm slices.

### fMRI data preprocessing

Functional data were preprocessed using Statistical Parametric Mapping 8 (SPM8) and movement corrected using ArtRepair (Mazaika et al., 2009). Resting data were collected during a 5 min run, resulting in 150 volumes of data. The first five volumes were removed from analyses to control for T1 equilibration effects, resulting in a time series of 145 volumes. All EPI data were slice-timing corrected, realigned to identify movement parameters for correction, coregistered to a high-resolution T1 image, normalized and smoothed using a 6 × 6 × 8 mm kernel.

#### Movement correction

The fMRI literature now recognizes the importance of addressing nuisance signal due to physiology and head motion (Power et al., 2012, 2014) and these issues are of particular relevance in studies of rsfMRI. To correct motion, raw data were examined for motion-related slice and volume signal changes using ArtRepair (Mazaika et al., 2009) including Artregress with 6 head movement parameters. Using this motion-correction pipeline reveals that in our TBI Time 1 group, where motion is typically the greatest concern, we can resolve motion problems in ~90% of the scans using standard ArtRepair guidelines (5% slice correction, 25% volume correction). Individuals above these thresholds were excluded. Global signal regression was not used during data processing (Murphy et al., 2009; Saad et al., 2012).

### Analytic approach and processing pipeline

**Figure 1:**
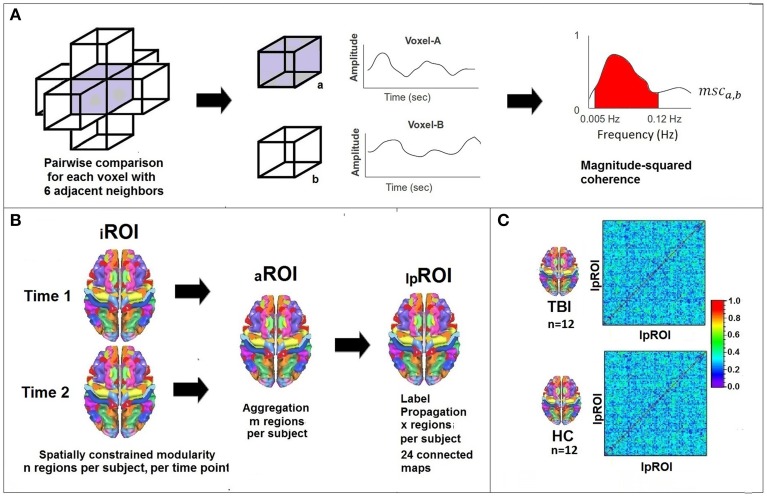
**Processing stream for data analysis. (A)** Lattice network permitting 6 adjacent possible links which are determined on a voxelwise basis using magnitude squared coherence in the frequency domain. fMRI signal filtered to analyze signal between 0.005 and 0.12 Hz. **(B)** Modularity constrained by 27 meta-AAL regions resulting in n regions per subject. Analyses conducted separately for Time 1 and Time 2 and then aggregated creating m regions. Label Propagation used to create a group-level map with x regions. **(C)** Schematic illustration of adjacency matrix (correlations between pairs of lpROIs) for the final graph-theoretic analysis of plasticity.

**Figure 2:**
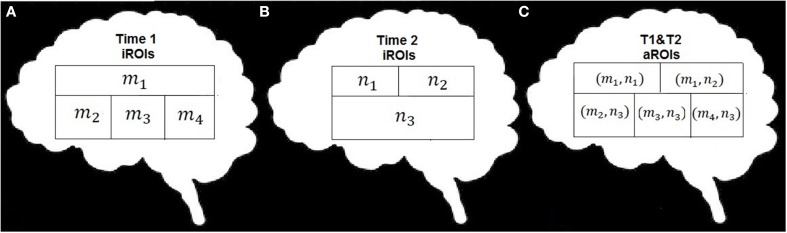
**Schematic illustration of the data combination**. **(A)** example data set with m nodes, **(B)** example data set with n nodes, **(C)** recombination of m x n nodes.

**Figure 3:**
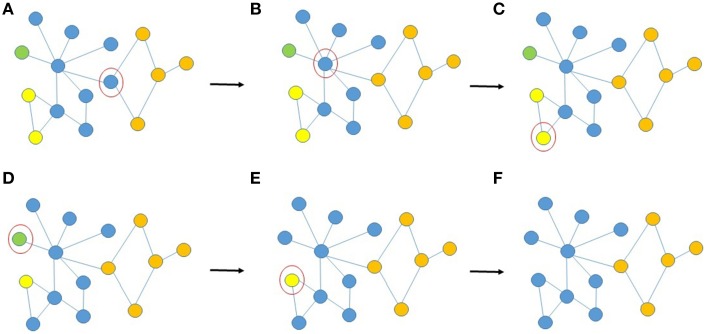
**Schematic illustration of the reassignment of voxel-nodes after fractionation of ROIs due to aggregation, (A–F) provides examples of AILP decisions during random selection of voxel-nodes (circled in red) for re-evaluation of group membership. (A)** Three of the voxel's neighbors are orange and one is blue, so it is assigned to the orange cluster, **(B)** five of the voxel's neighbors are blue, one is orange and one is green, it remains in the blue cluster, **(C)** one of the voxel's neighbors is yellow and one is blue (a tie), it is randomly assigned to one of the two clusters (let us assume blue). **(D/E)** the voxel's neighbors are blue, it is assigned to the blue cluster. **(F)** Convergence.

Figure 1 provides an overview of the analytic processing stream; there are three primary procedural steps to examine systems-level plasticity between time points. Table 2 provides definitions for the terminology used in describing the processing stream. First, whole-brain voxelwise analysis of community structure was performed using modularity (detail provided in section Determining ROIs of Functionally Connected Voxels Using Modularity) to arrive at individual time point ROIs (_i_ROIs). In this step, anatomically constrained analysis of voxelwise modularity was conducted in each individual at each time point resulting in 48 individual brain maps. The second analytic step was to conduct a spatial aggregation of the Time 1 and Time 2 _i_ROIs, resulting in an “aggregate map” of functionally connected ROIs, or _a_ROIs (see Figure 2). Finally, the AILP procedure was conducted to reconstitute the _a_ROIs, resulting in _lp_ROIs, which are the final set of regions used for the graph theoretical analysis of plasticity. Figure 3 provides a schematic representation of the label propagation procedure through several iterations of voxel reassignment to ameliorate the effects of ROI fractionation.

**Table 2:** **Definitions for terms used during analyses**.

Node	Representation of an individual unit within graph analysis. For our purposes, these can be voxel-nodes or ROI-nodes
_i_ROI	Collection of functionally coherent voxels at the individual level at a single time point; outcome of voxelwise modularity analysis
_a_ROI	Aggregation of two iROIs for each individual from Time 1 and Time 2. Results in a single map of aROIs for each subject
_lp_ROI	Final outcome of the AILP procedure, representing a recombination of the aROIs for each individual; final step in ROI creation
Edge	Representation of functional relationship between two graph components. For our purposes, edges exist between voxel-nodes or ROI-nodes
Network	Collection of voxel-nodes and edges as a large graph
Community Structure	The presence of subgroups of nodes within the graph which are densely connected internally but more sparsely connected between groups
Modularity	A measure of the presence of community structure in a network graph

#### Creation of the voxelwise brain network

*Binary gray-matter mask:* For each subject, we first developed an average functional image using all 145 volumes and then coregistered their T1 gray matter segmented image to the average functional image. The coregistered T1 images were averaged over subjects and then converted to a binary image using imcalc (SPM8) with a threshold of 0.5. The binary mask was developed using Time 1 data and then used at both time points. Creation of a binary mask permitted equivalence in the voxels submitted to whole brain analyses for longitudinal comparison.

#### Lattice representation of voxelwise adjacencies

We examine whole-brain voxelwise connectivity using functionally defined connections with spatial constraints. In particular, a lattice network was created for all voxels within the gray matter mask so that each voxel was represented as a node in the representative network graph and was connected with an edge to each of its adjacent 6 neighbors, provided these neighbors were members of a larger spatially-constrained anatomical map based upon 27 “meta-regions” using the Automated Anatomic Labeling (AAL) atlas (see Table 3) (Tzourio-Mazoyer et al., 2002). That is, edges were not considered between adjacent voxels across the boundaries of the 27 meta-regions. Let us denote the resulting network graph *G* = (*V*, *E*) where *V* is the set of voxel-nodes and *E* the set of edges between node pairs. Furthermore, let |*V*| = *n* and |*E*| = *m*.

**Table 3:** **Meta-AAL assignment and anatomical landmarks for parcellation**.

**Meta region(s)**	**Anatomical landmark; AAL Number**
1–2	Frontal Lobe L/R; AAL: 1–28
3–4	Insula L/R; AAL: 29–30
5–6	Occipital Lobe L/R; AAL: 43–56
7–8	Parietal Lobe L/R; AAL: 57–70
9–10	Thalamus, L/R; AAL: 111–112
11–12	Temporal L/R; AAL: 79–90
13–14	Cerebellum; AAL: 91–116
15	Vermis of Cerebellum, AAL: 109–116
16–17	Anterior and middle cingulate cortex, AAL: 31–34
18–19	Posterior cingulate cortex L/R; AAL: 35–36
20–21	Hippocampus-Amygdala L/R; AAL: 37–42
22–23	Caudate nucleus L/R; AAL 71–72
24–25	Putamen L/R; AAL 73–74
26–27	Globus Pallidus L/R; AAL 75–76

L, left; R, right; for complete AAL list see Supplemental Table 1.

We determine a weight on each edge present in the lattice network using the low frequency (0.005–0.12 Hz; Bassett et al., 2011) coherence between the signals representing the voxels that form the edge. To accomplish this we first calculate the magnitude square coherence (MSC) between a pair of signals (See Figure 1A) using multi-taper technique (Thomson, 1982; Babadi and Brown, 2014) and then evaluate the area under the MSC-spectrum between 0.005 and 0.12 Hz. Thus high area under the curve would suggest that the signals representing the voxels are highly coherent in this given low-frequency band.

#### Determining ROIs of functionally connected voxels using modularity

*Modularity*: Modularity is a measure of community structure borrowed from network science and has been applied to examine large-scale network changes in brain diseases such as multiple sclerosis (Gamboa et al., 2014), schizophrenia (Yu et al., 2012), traumatic brain injury (Han et al., 2014) and neurodegenerative disorders such as Parkinson's Disease (Baggio et al., 2014) and AD (Brier et al., 2014). For our purposes, modules (densely connected groups) of voxel-nodes (simplified as “node” here) can be interpreted as functionally meaningful _i_ROIs. The modularity *Q* of a graph can be summarized computationally as:

$$Q=\frac{1}{2m}\sum_{ij}\left(A_{ij}-\frac{k_{i}k_{j}}{2m}\right)\delta \left(C_{i},C_{j}\right).$$

Recall, *m* is the number of edges in the graph. For node *i* and node *j* in the network, *A*_*ij*_ represents the *ij*-entry in the corresponding graph adjacency matrix, *k*_*i*_ is the degree of node *i* calculated as the sum of the weights on all edges incident to node *i*, and δ(*C*_*i*_, *C*_*j*_) is the Kronecker delta function, which is equal to 1 if node *i* is in module *C*_*i*_ and node *j* is in *C*_*j*_, and 0 otherwise. In this way, *Q* can be interpreted as a fitness measure of a given partition of the network into communities *C*_*i*_ where greater *Q* indicates a stronger partition, or more edges within communities and fewer edges between communities (Clauset et al., 2004). In the weighted formulation of this, we seek to find a partition of the network graph into communities such that the total weight of all edges that fall within modules is greater than the expected total weight of within-module edges for a comparable network with the same degree distribution but whose edge weights are distributed at random. The set of modules *C*_*i*_ determined to maximize *Q* is taken as the set of _i_ROIs. We implement the Louvain modularity optimization algorithm (Blondel et al., 2008) over our weighted, undirected lattice graph for each individual (24) at each time point (2) resulting in 48 distinct partitions of the network into modules at each individual time point, or 48 sets of _i_ROIs.

#### Aggregation

We adapt procedures of label propagation and module aggregation proposed in (Raghavan et al., 2007) to fit the specific features of the fMRI datasets. As noted above we create a representative functional network with spatial (anatomical) constraints which was not a feature during development of the original label propagation algorithm. For each subject, the _i_ROIs determined using whole-brain spatially-constrained modularity optimization at each time point were spatially aggregated by assigning each voxel i an ordered pair label (*m*_*i*_, *n*_*i*_) (one for Time 1 and one for Time 2) and joining all voxels with the same label into one module (see Figure 2). Accordingly, the aggregation procedure can be interpreted as determining the intersection of the parts of the network partitions under investigation (in our case Time 1 and Time 2). The result of aggregation is a single consensus set of modules (aggregated ROIs, or _a_ROIs) for each subject.

#### Label propagation

One undesirable feature of aggregation is that it commonly results in fractionation of the _i_ROIs, resulting in very small regions (e.g., 1–2 voxels) identified as independent _a_ROIs when two very similar but not identical regions are overlapped (see Figure 3). In practice, if the voxel-nodes composing these small fractionated _a_ROIs have more neighbors in a different _a_ROI than in their own we allow them to re-affiliate using label propagation (Raghavan et al., 2007). The label propagation algorithm is an iterative procedure outlined as follows.

1. For a given voxel *i*, let *M*_*i*_ be the module affiliation of *i* after aggregation. Initialize *C*_*i*_(0) = *M*_*i*_.
2. Set *t* = 1.
3. Arrange all voxels in the network in a random order and store this order as *X*.
4. For each *i* ∈ *X*, selected in order, let
  $$\begin{array}{l} C_{i}\left(t\right)=f(C_{j1}\left(t\right),\ldots ,C_{jm}\left(t\right),C_{j(m+1)}\left(t-1\right),\ldots ,\\ \;C_{jk}\left(t-1\right)), \end{array}$$
  where f here returns the label occurring with the highest frequency among the neighbors of *i*, (*j*_1_, …, *j*_*k*_), and ties are broken uniformly randomly.
5. If every voxel-node has the label that the maximal number of its neighbors have, the algorithm concludes. Else, set *t* = *t* + 1 and go to (3).

The outcome of this procedure is a new set of label-propagated ROIs, or _lp_ROIs, which are the focus of graph theoretical analysis in the final step.

#### Graph theoretical analysis of network plasticity

Following the AILP procedure, final time-consistent _lp_ROIs were used for graph-theoretic analysis of network plasticity. For each subject, for each time point separately, the mean fMRI signal for each of the common _lp_ROIs was correlated with each other and an adjacency matrix for all pairwise correlations amongst all _lp_ROIs was created. In this sense, the resulting graph is a meta-network representation of the original network considered in Steps 1–3 composed of ROI-nodes that are the result of the AILP (_lp_ROI).

Based upon Pearson correlation value (thresholded using false discovery rate at *p* < 0.05), _lp_ROIs with statistically significant correlations were joined by a weighted link in the representative network, where weights were determined as the value of the corresponding correlations (Bassett et al., 2011). Graph metrics studied over this network included: (1) total number of links, (2) total network strength (sum of the weights for all links), (3) clustering coefficient (defined below), and (4) path length (defined below).

The mean clustering coefficient for a network *C*, is given by $\bar{C}=\frac{1}{n}\sum_{i\;=\;1}^{n}C_{i}$, where

$$C_{i}=\frac{2|\{e_{jk}:j,k\in N_{i},e_{jk}\in E\}|}{k_{i}(k_{i}-1)}$$

and *N*_*i*_ is the neighborhood of *i*.

Path length was determined using binary connections (unweighted edges) given the difficulty in interpreting weighted path length in functional brain networks. The mean unweighted path length for a network *l*_*G*_, is determined as $l_{G}=\frac{1}{n(n-1)}\sum_{i\;\neq \;j}d(i,j)$, where *d*(*i*, *j*) is the shortest distance (i.e., the number of edges in the shortest path) between node *i* and node *j*.

## Results

### Modular structure of the brain

Whole-brain modularity analysis conducted for all subjects revealed avrelatively consistent number of brain network communities. The mean number of _i_ROIs and voxels per _i_ROI for each group and time point are presented in Table 4.

**Table 4:** **Parcellation results for modularity, aggregation, and AILP for TBI sample**.

	**Time 1 (iROI)**	**Time 2 (iROI)**	**Aggregation only (aROI)**	**Aggregation + Label Propagation (lpROI)**
Total number of modules (ROIs)	343.7 (123.6)	289.1 (5.4)	921.67 (129.8)	509.3 (19.0)
Voxel count/ module: Mean/(sd)/median	86.1 (62.2) 79	86.3 (63.4) 79	27.2 (25.2) 12	47.1 (44.48) 32
% of ROIs with <5 voxels	2.4%	2.9%	33.6%	8.1%
% of ROIs with <10 voxels	7%	7.8%	47.1%	18.9%

### Aggregation of data during overlay

In order to examine change between time points in this sample of individuals with TBI, an aggregation of the data is required. This step overlaid the _i_ROI partition results for each time point, resulting in _i_ROI fractionation and a significant increase in the number of total regions, including regions that were anatomically unorthodox and/or very small (~1–5 voxels). Table 4 reveals the increase in the number of regions during the aggregation step and mean, standard deviation and median for voxel counts per ROI during each step of the analysis.

### Label propagation of aggregated data

**Figure 4:**
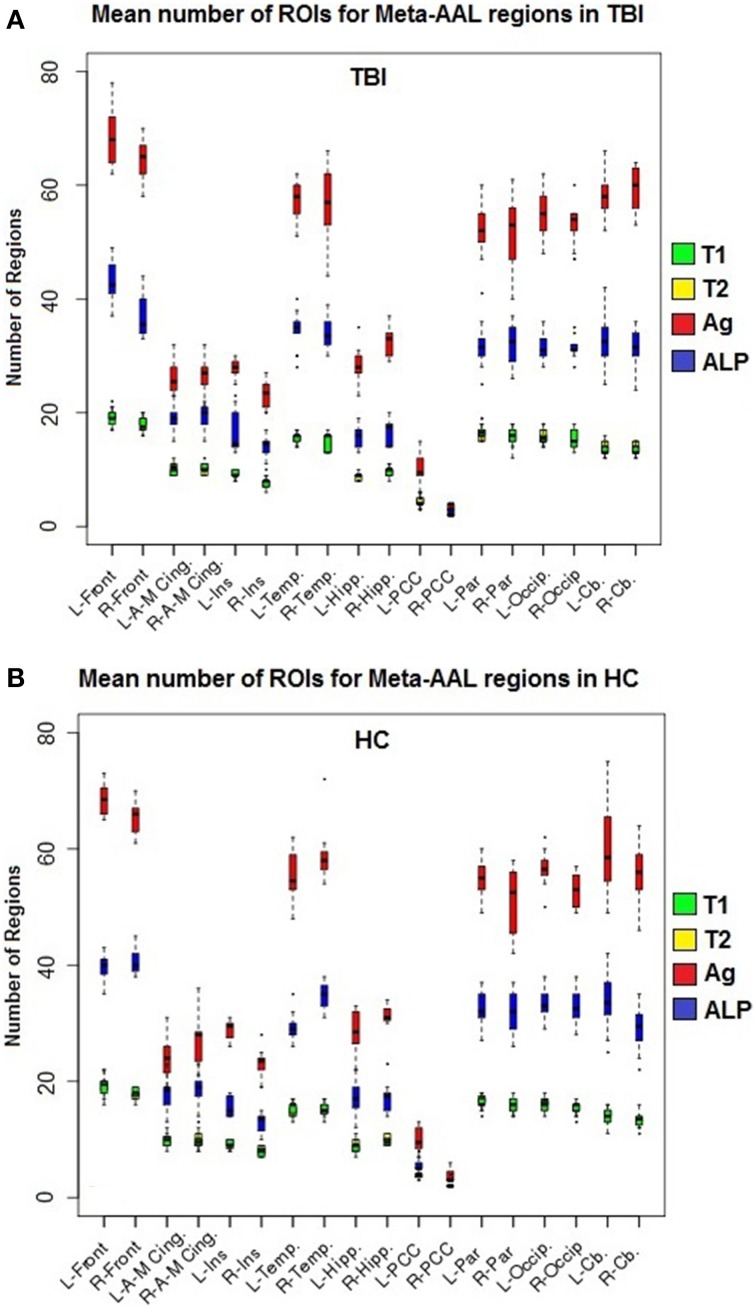
**Box plots of the mean number of ROIs for each meta-region. (A)** Healthy control subjects, **(B)** TBI subjects; T1, Time 1 whole-brain modularity results (green); T2, Time 2 whole-brain modularity results; Ag, Aggregation of Time 1 and Time 2 (red); and ALP, Aggregation + Label propagation (blue). Abbreviations: A-M, anterior to middle; Cing, cingulate; Front, Frontal; Hipp, hippocampus; Ins, insula; L, left; Occip, occipital; Par, parietal; PCC, posterior cingulate cortex; R, Right, Temp, Temporal lobe.

**Figure 5:**
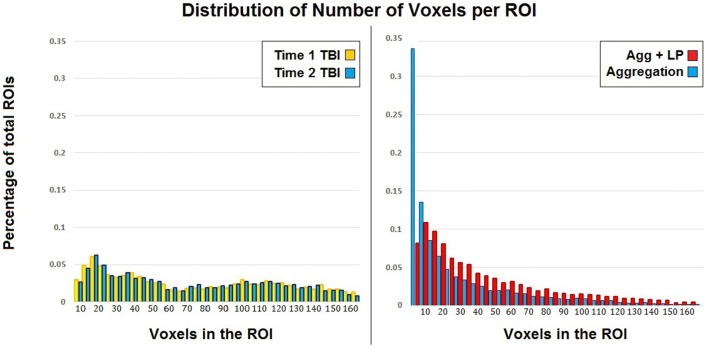
**Distribution of the voxels in each ROI for each time point and after aggregation only and AILP in the TBI sample**.

**Figure 6:**
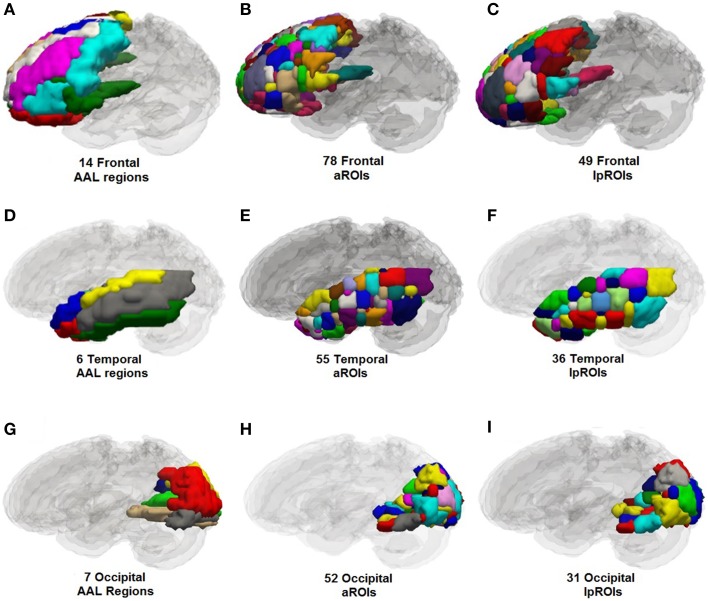
**Anatomical plots of original anatomical atlas (AAL), parcellations for the aggregation alone and aggregation + label propagation steps for frontal, temporal, and occipital lobes**. Parcellation values are for left hemisphere only. **(A)** AAL frontal, **(B)** aROIs frontal, **(C)** lpROIs frontal. **(D)** AAL temporal, **(E)** aROIs temporal, **(F)** lpROIs temporal. **(G)** AAL occipital, **(H)** aROIs occipital, **(I)** lpROIs occipital.

Label propagation was performed in order to reconstitute _a_ROIs into functionally homogeneous _lp_ROIs while preserving the original data at the voxel level for each time point. Figure 4 show box plots for the number of each type of ROI at each step of the analysis. As expected, Time 1 and Time 2 data reveal fewer _i_ROIs separately for both samples. The aggregated data show a dramatic increase in the number of regions and label propagation consistently reduces the number of regions based upon the aggregation-only step. Table 4 reveals the increase in the number of regions during the aggregation and median voxel count before and after label propagation. Figure 5 indicates the distribution of the number of voxels in each ROI before and after label propagation for all TBI subjects. The distributions reveal a decrease in the number of regions with fewer than 5 voxels (32% of total during aggregation, 8% of total after AILP). See Figure 6 for a spatial representation of the final aggregation and label propagation steps for frontal, temporal and occipital cortices.

### Robustness of AILP

It is a goal for any ROI selection method to be robust to minor changes in parameter selection and, in the case of a method such as ours which includes a step with stochasticity, consistent across runs over the same dataset.

The aggregation and label propagation procedure we have proposed involves few investigator-tuned parameters. Aggregation is achieved through computing intersections between the _i_ROI maps at each time point, which is a deterministic process requiring no input parameters. The label propagation procedure involves one investigator decision, namely the choice of neighborhood criteria. As iterations of LP unfold, the membership of each voxel-node is assessed as the majority ROI assignment of its neighbors, requiring a formal definition of which voxels should be considered neighboring. We implement a lattice graph definition of adjacency, whereby each voxel is assumed to neighbor six contiguous voxels. The lattice structure we use here is chosen to be consistent with the lattice graph we use in Step 1 of our described algorithm (see Section Creation of the Voxelwise Brain Network) for determining _i_ROIs at each time point with modularity optimization. However a lattice is not required, and both modularity and AILP could be applied to differently organized graph structures (e.g., allow each voxel to consider neighbors within a given spatial radius) if an investigator has cause for this type of analysis.

The most significant consideration with respect to the reliability of AILP over multiple runs are two points of stochasticity in the algorithm. First, at each iteration of the procedure, each voxel-node is examined to determine possible reassignment to a new _lp_ROI (see Figure 3). The order in which voxels are selected for this process is randomly generated at each iteration (see Step 3 of the pseudocode in Section Determining ROIs of Functionally Connected Voxels Using Modularity). Secondly, in the case in which a voxel determines that an equal number of its neighbors participate in more than one _lp_ROI (i.e., there is a tie), that voxel will be assigned to an _lp_ROI at random (see Step 4 of the pseudocode in Section Determining ROIs of Functionally Connected Voxels Using Modularity) (Figure 3C). Because of these two sources of variability, distinct runs of the LP algorithm could theoretically result in distinct voxel-to-_lp_ROI assignments. To investigate whether these differences in the procedure may result in distinct sets of final _lp_ROIs over multiple runs on the same dataset, we perform 10 runs of the LP procedure for two randomly selected subjects (one from the HC sample and one TBI sample for completeness) and compare results.

The Sørensen similarity index (Sørensen, 1948) is one well-established metric for quantifying the consistency of a pair of samples, *X* and *Y*. It is defined $s_{XY}=\frac{2|X\cap Y|}{\left|X\right|+|Y|}$. We calculate *s*_*XY*_ for each pair of _lp_ROIs (here, *X* and *Y*) in each meta-AAL region, for each of the 45 possible pairwise combinations of the 10 runs (runs 1 and 2, runs 1 and 3, runs 1 and 4, etc.). To capture the overall consistency of ROIs for each subject, we report the average of these scores overall _lp_ROIs, weighted by the size of the _lp_ROI. The healthy control and TBI subjects report 95.8% and 96.4% consistency, respectively, over the 45 pairs of runs.

As an additional analysis of _lp_ROI reliability, we measure the probability that any pair of voxels will be consistently assigned to the same _lp_ROI. Specifically, for each pair of voxels in each meta-AAL region, for each of the 10 runs we examine the probability of consistent assignment (10 consistent assignments = 100%, 9 consistent assignments = 90%, etc.). For this analysis, the normalized average score for voxel-pair-wise reliability was 95.6 and 96.2% for the healthy control and TBI subjects, respectively.

### Graph analysis of plasticity using AILP-derived ROIs

To provide context for how _lp_ROIs might be used in the study of plasticity, all _lp_ROIs for each individual were entered into a graph for analysis. Table 5 reveals the results for global graph metrics in the TBI and HC samples. These metrics are based upon a graph theoretical analysis using Pearson's correlation as the determinant for relationships between _lp_ROI-nodes. The TBI sample demonstrates increased connectivity during recovery in several metrics including total *number of links* and *network strength*. During this recovery window, community structure also changes, as the network shows a higher mean *clustering coefficient*.

**Table 5:** **General graph properties in TBI and HC groups**.

	**TBI Time 1 Mean (*sd*)**	**TBI Time 2 Mean (*sd*)**	**HC Combined Mean (*sd*)**
Total number of connections	75,975.8^*^ (19,184.5)	90,829.7^*^ (17,924.9)	80,844.67 (27,383)
Total strength of connections	34,093.9^*^ (10,645.6)	42,470.22^*^ (11,230.7)	37,186.7 (16,238.04)
Average path length	1.388^*^ (0.171)	1.288^*^ (0.15)	1.378 (0.204)
Mean clustering coefficient	0.375^*^ (0.074)	0.424^*^ (0.068)	0.385 (0.092)

Within-group comparison:

* p < 0.05;

differences between TBI sample and HC sample non-significant. Note: no significant differences between Time1 and Time 2 HC for any graph metrics, therefore mean data presented; sd, standard deviation.

## Discussion

The current study presents a reliable approach to determine meaningful brain regions for the study of network plasticity. The approach leverages the label propagation algorithm for community structure detection, initialized using an aggregation of functional brain regions generated at each time point. There are two primary discussion points from this investigation that require elaboration, the first is the validation of the AILP procedure and the second is its application to the study of plasticity in TBI. With regard to the first, given a set of functionally discrete nodes, the AILP algorithm is capable of aggregating covarying but spatially distinct signals, affording a single representative map of functional organization. A test of the reliability of repeat measurements of the AILP revealed ~95% spatial overlap in the _lp_ROIs and significant reduction in the number of small or isolated ROIs created during aggregation. This reliability evident during both voxel-based and ROI-based measurements. Specifically, in the absence of label propagation, the aggregation of Time 1 and Time 2 data resulted in a 268 and 318% increase, respectively, in the number of regions as compared to the individual time points and most importantly, nearly 1/3 of the regions created during the aggregation were less than 5 voxels in size, compared to 2–3% for the TBI data at individual time points. It is possible that some of these very small identified regions were functionally distinct units (several subcortical regions have <10 voxels). However given that we used a standard smoothing kernel (6 × 6 × 8 mm) during preprocessing, there is a significantly correlation between adjacent voxels and given that most clustering correction methods require >20 contiguous voxels (Saad and Reynolds, 2012), the meaningfulness of regions consisting of <5 voxels is uncertain. Following the label propagation, the total number of _lp_ROIs fewer than 5 voxels was at 8% (see Figure 5, Table 4). Of particular importance, the AILP algorithm achieved this ROI reconfiguration without supervision or thresholding. Investigators aiming to further limit the number of very small ROIs from analysis could do so with a clustering filter post-AILP, for example using a similar approach to maximize modularity over clusters of smaller regions. To demonstrate the power of AILP we took none of those steps, but they would be natural modifications to this approach requiring minimal supervision. In sum, we anticipate that the result of the AILP approach is a homogeneous and functionally discrete set of _lp_ROIs that can reliably be used for secondary analyses of network plasticity.

It should be noted that even with the AILP procedure, there is a significant increase in the number of _lp_ROIs compared to either Time 1 or Time 2 data in isolation (Table 4). Of course, in the absence of perfect overlap for all ROIs, any aggregation procedure will produce an increase in the number of nodes; the question is: at what threshold is this increase problematic? In the absence of a gold standard we look to two factors to guide data interpretation. First, we look to the relative size of the ROI-nodes as an important indicator of ROI viability and the reliable convergence of the AILP algorithm indicating that a solution was achievable. Because of the increased likelihood of very small _lp_ROIs being the result of statistical artifact or side-effects of pre-processing (e.g., spatial smoothing) very small ROIs are more likely to be spurious (e.g., fewer than 5–10 voxels). This is where we see the greatest reconfiguration by the AILP algorithm, reconstituting many of the very small _lp_ROIs so that the number of _lp_ROIs with less than 10 voxels diminishes from 47% during aggregation to 18% after AILP. Second, because the AILP functions to align ROIs by clustering nearest neighbors, it prohibits two phenomena that are likely to result in statistically or neuroanatomically spurious partitions: (1) individual “lone” voxels represented as distinct ROIs, (2) partitions with irregular geometric shapes—i.e., non-contiguous, doughnut-shaped, or otherwise anatomically spurious. Therefore, using a recurrent lattice-like array, the AILP procedure is consistent with a neuroanatomical structure that has been repeatedly shown to be a foundational principle of cortical organization (see Rockland and Lund, 1983; Rockland, 1985; McGuire et al., 1991; Lund et al., 1993). To the degree that brain organization has scale free properties, these seminal anatomical studies provide some reassurance that using a lattice network as the basis for the current AILP procedure is implementing anatomically plausible partitions. Thus, in this study, the modularity step guarantees that regions are coherent with respect to signal and the AILP step enforces spatial constraints during recombination.

In the current study, the first step in devising _i_ROIs for each time point makes use of modularity given its widespread success in determining community structure for large, complex networks in various domains. Modularity maximization algorithms permit completely unsupervised data parcellation with computational efficiency, an important consideration for a 26 × 26 k matrix. Modularity therefore was a conservative choice for creating single time point _i_ROIs to submit to the AILP procedure, however, this first step could include any of the available parcellation methods. Moreover, the AILP procedure was adapted to make use of the original voxel assignment, but is not influenced by the nature of the signal during its re-partitioning. So while we focus on voxel-level analyses in this application, the AILP is agnostic about the nature of the spatially constrained data that is used as input and could be applied to other functional data subtypes or even structural or anatomical data wherein subtle changes may be expected over time resulting in reconstitution of its elements.

The second point of discussion regarding the AILP procedure is that the fMRI ROIs determined using AILP retain their native signal permitting flexibility for further analysis, including the homogeneity of the signal in each region or even secondary analysis of the intra-regional connectivity between voxels. In the service of studying network plasticity, the signal from _lp_ROIs in the current study were averaged and entered into a graph-theoretical analysis to examine global network changes from time 1 to time 2. The increased connectivity detected in the TBI sample during recovery is consistent with analysis of this dataset focusing on regions with specific sub-networks (e.g., posterior cingulate cortex, medial frontal cortex) (see Venkatesan et al., 2014) and the literature more broadly (Castellanos et al., 2010; Bonnelle et al., 2011; Hillary et al., 2011a,b, 2015; Sharp et al., 2011; Caeyenberghs et al., 2012). What the AILP approach affords is sensitivity to whole-brain, voxelwise change in network topology.

### Limitations and future directions

The primary advancement here is that the aggregation and label propagation procedure permits the investigator to find a consensus set of brain regions across time points. There are limitations to this approach that require mention. First, for fMRI the AILP is ideally used when there is some spatial information regarding voxel status. We implement a lattice to determine adjacencies which carries assumptions regarding voxelwise relationships (e.g., excluding diagonal neighbors), but this could also be represented in other ways including larger voxel neighborhoods or by inputting physical connections in a structural graph. Also, with regard to our example for application to graph theory, we interpret the findings with respect to connection loss and gain in TBI as an initial step in understanding the consequences of injury; the edges represent only a partial approximation of the underlying connectivity (for review see Rockland, 2015). While it is outside the scope of the current study where we focus on validation of the AILP, continued testing of the *hyperconnectivity hypothesis* requires more detailed analysis including tests of the non-stationarity in the connections and additional measures of the physical connectedness including physical distance as a marker for connection cost and efficiency.

In summary, we offer an efficient and mathematically tractable method for voxelwise concatenation of distinct fMRI datasets in multiple time points. The AILP approach is ideal for studies focused on developmental processes, as demonstrated in the current example to document changes in whole-brain networks after traumatic brain injury including a shift toward hyperconnectivity.

### Conflict of interest statement

The authors declare that the research was conducted in the absence of any commercial or financial relationships that could be construed as a potential conflict of interest.
