# Supplementary material for: A voxelwise approach to determine consensus regions-of-interest for the study of brain network plasticity
Source: Front Neuroanat. 2015 Jul 28;9:97. doi: 10.3389/fnana.2015.00097 (PMC4517380; doi:10.3389/fnana.2015.00097)
Supplement: Supplementary file 1 [file DataSheet1.DOCX]

Supplemental Table 1: Complete list of AAL Atlas regions and assignment to meta-AAL regions for the study:

Tzourio_Mazoyer_ID|Brain_region_name|WOROI|meta

1|Left_precentral_gyrus|144|1

2|Right_precentral_gyrus|145|2

3|Left_superior_frontal_gyrus,_dorsolateral|146|1

4|Right_superior_frontal_gyrus,_dorsolateral|147|2

5|Left_superior_frontal_gyrus,_orbital_part|690|1

6|Right_superior_frontal_gyrus,_orbital_part|691|2

7|Left_middle_frontal_gyrus,_lateral_part|696|1

8|Right_middle_frontal_gyrus,_lateral_part|697|2

9|Left_middle_frontal_gyrus,_orbital_part|699|1

10|Right_middle_frontal_gyrus,_orbital_part|700|2

11|Left_opercular_part_of_inferior_frontal_gyrus|670|1

12|Right_opercular_part_of_inferior_frontal_gyrus|671|2

13|Left_area_triangularis|678|1

14|Right_area_triangularis|679|2

15|Left_orbital_part_of_inferior_frontal_gyrus|681|1

16|Right_orbital_part_of_inferior_frontal_gyrus|682|2

17|Left_rolandic_operculum|675|1

18|Right_rolandic_operculum|676|2

19|Left_supplementary_motor_area|199|1

20|Right_supplementary_motor_area|200|2

21|Left_olfactory_cortex|687|1

22|Right_olfactory_cortex|688|2

23|Left_superior_frontal_gyrus,_medial_part|701|1

24|Right_superior_frontal_gyrus,_medial_part|702|2

25|Left_superior_frontal_gyrus,_medial_orbital_part|693|1

26|Right_superior_frontal_gyrus,_medial_orbital_part|694|2

27|Left_gyrus_rectus|156|1

28|Right_gyrus_rectus|157|2

29|Left_insula|120|3

30|Right_insula|121|4

31|Left_anterior_cingulate_gyrus|94|3

32|Right_anterior_cingulate_gyrus|95|4

33|Left_middle_cingulate|158|3

34|Right_middle_cingulate|159|4

35|Left_posterior_cingulate_gyrus|3

36|Right_posterior_cingulate_gyrus|4

37|Left_hippocampus|107|3

38|Right_hippocampus|108|4

39|Left_parahippocampal_gyrus|131|3

40|Right_parahippocampal_gyrus|132|4

41|Left_amygdala|98|3

42|Right_amygdala|99|4

43|Left_calcarine_sulcus|162|5

44|Right_calcarine_sulcus|163|6

45|Left_cuneus|164|5

46|Right_cuneus|165|6

47|Left_lingual_gyrus|166|5

48|Right_lingual_gyrus|167|6

49|Left_superior_occipital|174|5

50|Right_superior_occipital|175|6

51|Left_middle_occipital|176|5

52|Right_middle_occipital|177|6

53|Left_inferior_occipital|179|5

54|Right_inferior_occipital|18|6

55|Left_fusiform_gyrus|133|5

56|Right_fusiform_gyrus|134|6

57|Left_postcentral_gyrus|184|7

58|Right_postcentral_gyrus|185|8

59|Left_superior_parietal_lobule|186|7

60|Right_superior_parietal_lobule|187|8

61|Left_inferior_parietal_lobule|188|7

62|Right_inferior_parietal_lobule|189|8

63|Left_supramarginal_gyrus|190|7

64|Right_supramarginal_gyrus|191|8

65|Left_angular_gyrus|192|5|7

66|Right_angular_gyrus|193|6|8

67|Left_precuneus|194|7

68|Right_precuneus|195|8

69|Left_paracentral_lobule|197|7

70|Right_paracentral_lobule|198|8

71|Left_caudate_nucleus|118|9

72|Right_caudate_nucleus|119|10

73|Left_putamen|116|9

74|Right_putamen|117|10

75|Left_globus_pallidus|114|9

76|Right_globus_pallidus|115|10

77|Left_thalamus|111|9

78|Right_thalamus|112|10

79|Left_transverse_temporal_gyri|264|11

80|Right_transverse_temporal_gyri|265|12

81|Left_superior_temporal_gyrus|129|11

82|Right_superior_temporal_gyrus|130|12

83|Left_superior_temporal_pole|269|11

84|Right_superior_temporal_pole|270|12

85|Left_middle_temporal_gyrus|266|11

86|Right_middle_temporal_gyrus|267|12

87|Left_middle_temporal_pole|271|11

88|Right_middle_temporal_pole|272|12

89|Left_inferior_temporal_gyrus|152|11

90|Right_inferior_temporal_gyrus|153|12

91|Left_crus_I_of_cerebellar_hemisphere|730|13

92|Right_crus_I_of_cerebellar_hemisphere|731|14

93|Left_crus_II_of_cerebellar_hemisphere|733|13

94|Right_crus_II_of_cerebellar_hemisphere|734|14

95|Left_Lobule_III_of_cerebellar_hemisphere|721|13

96|Right_Lobule_III_of_cerebellar_hemisphere|722|14

97|Left_lobule_IV,_V_of_cerebellar_hemisphere|724|13

98|Right_lobule_IV,_V_of_cerebellar_hemisphere|725|14

99|Left_Lobule_VI_of_cerebellar_hemisphere|727|13

100|Right_Lobule_VI_of_cerebellar_hemisphere|728|14

101|Left_lobule_VIIB_of_cerebellar_hemisphere|736|13

102|Right_lobule_VIIB_of_cerebellar_hemisphere|737|14

103|Left_lobule_VIII_of_cerebellar_hemisphere|739|13

104|Right_lobule_VIII_of_cerebellar_hemisphere|740|14

105|Left_lobule_IX_of_cerebellar_hemisphere|742|13

106|Right_lobule_IX_of_cerebellar_hemisphere|743|14

107|Left_lobule_X_of_cerebellar_hemisphere_(flocculus)|745|13

108|Right_lobule_X_of_cerebellar_hemisphere_(flocculus)|746|14

109|Lobule_I,_II_of_vermis|712|15

110|Lobule_III_of_vermis|713|15

111|Lobule_IV,_V_of_vermis|714|15

112|Lobule_VI_of_vermis|715|15

113|Lobule_VII_of_vermis|716|15

114|Lobule_VIII_of_vermis|717|15

115|Lobule_IX_of_vermis|718|15

116|Lobule_X_of_vermis_(nodulus)|719|15
